# Harnessing transcriptomic signals for amyotrophic lateral sclerosis to identify novel drugs and enhance risk prediction

**DOI:** 10.1016/j.heliyon.2024.e35342

**Published:** 2024-07-29

**Authors:** Oliver Pain, Ashley Jones, Ahmad Al Khleifat, Devika Agarwal, Dzmitry Hramyka, Hajer Karoui, Jędrzej Kubica, David J. Llewellyn, Janice M. Ranson, Zhi Yao, Alfredo Iacoangeli, Ammar Al-Chalabi

**Affiliations:** a Maurice Wohl Clinical Neuroscience Institute, Department of Basic and Clinical Neuroscience, Institute of Psychiatry, Psychology and Neuroscience, King's College London, London, United Kingdom; b Wellcome Centre for Human Genetics, Nuffield Department of Medicine, Old Road Campus, University of Oxford, Oxford, United Kingdom; c Core Unit Bioinformatics (CUBI), Berlin Institute of Health, Charité – Universitätsmedizin Berlin, Berlin, Germany; d Max Delbrück Center for Molecular Medicine in the Helmholtz Association, Berlin, Germany; e Multiple Sclerosis and Parkinson's Tissue Bank, Department of Brain Sciences, Imperial College London, London, United Kingdom; f Laboratory of Structural Bioinformatics, Institute of Evolutionary Biology, University of Warsaw, Poland; g Laboratory of Theory of Biopolimers, Faculty of Chemistry, University of Warsaw, Poland; h University of Exeter Medical School, Exeter, United Kingdom; i Alan Turing Institute, London, United Kingdom; j LifeArc, Stevenage, United Kingdom; k Department of Biostatistics and Health Informatics, Institute of Psychiatry, Psychology & Neuroscience, King's College London, London, United Kingdom; l National Institute for Health Research Biomedical Research Centre and Dementia Unit at South London and Maudsley NHS Foundation Trust and King's College London, London, United Kingdom

## Abstract

**Introduction:**

Amyotrophic lateral sclerosis (ALS) is a fatal neurodegenerative disease. This study integrates common genetic association results from the latest ALS genome-wide association study (GWAS) summary statistics with functional genomic annotations with the aim of providing mechanistic insights into ALS risk loci, inferring drug repurposing opportunities, and enhancing prediction of ALS risk and clinical characteristics.

**Methods:**

Genes associated with ALS were identified using GWAS summary statistic methodology including SuSiE SNP-based fine-mapping, and transcriptome- and proteome-wide association study (TWAS/PWAS) analyses. Using several approaches, gene associations were integrated with the DrugTargetor drug-gene interaction database to identify drugs that could be repurposed for the treatment of ALS. Furthermore, ALS gene associations from TWAS were combined with observed blood expression in two external ALS case-control datasets to calculate polytranscriptomic scores and evaluate their utility for prediction of ALS risk and clinical characteristics, including site of onset, age at onset, and survival.

**Results:**

SNP-based fine-mapping, TWAS and PWAS identified 118 genes associated with ALS, with TWAS and PWAS providing novel mechanistic insights. Drug repurposing analyses identified six drugs significantly enriched for interactions with ALS associated genes, though directionality could not be determined. Additionally, drug class enrichment analysis showed gene signatures linked to calcium channel blockers may reduce ALS risk, whereas antiepileptic drugs may increase ALS risk. Across the two observed expression target samples, ALS polytranscriptomic scores significantly predicted ALS risk (*R*^2^ = 5.1 %; *p*-value = 3.2 × 10^−27^) and clinical characteristics.

**Conclusions:**

Functionally-informed analyses of ALS GWAS summary statistics identified novel mechanistic insights into ALS aetiology, highlighted several therapeutic research avenues, and enabled statistically significant prediction of ALS risk.

## 1 Introduction

Amyotrophic lateral sclerosis (ALS), also known as motor neurone disease, is a neurodegenerative disease affecting both upper and lower motor neurones, leading to progressive loss of voluntary muscle control, and ultimately respiratory failure within 3–5 years after disease onset [1]. The lifetime risk of ALS is 1 in 300 and a mean age of onset of 56 years, making it the most common neurodegenerative midlife disease [2,3]. There is no diagnostic test for ALS, and substantial heterogeneity in clinical presentation and speed of progression make diagnosis of ALS challenging [4]. ALS is currently diagnosed based on a series of tests ruling out other diseases, alongside a detailed history of symptoms observed by a physician, and a full medical history. The U.S. Food and Drug Administration has currently approved three drugs for the treatment of ALS, including Riluzole, Edavarone and Relyvrio. However, these drugs have a limited effect on life expectancy and daily functioning [[5], [6], [7]]. Further development of pharmacological treatments for ALS is therefore needed.

There is substantial evidence that genetic factors play an important role in the aetiology of ALS. Between 5 and 20 % of people with ALS have a clear family history, referred to as familial ALS, with non-familial cases of ALS referred to as sporadic ALS [8]. Within familial cases of ALS, several rare genetic variants with large effect on ALS risk have been identified, such as within genes *C9orf72* [9] and *TBK1* [10]. Sporadic ALS cases also have a strong genetic aetiology, with twin-based heritability estimates between 40 and 60 %, and ∼20 % of those with the sporadic disease carrying known ALS risk variants [[11], [12], [13]]. In addition to the rare large effect variants associated with ALS, there is evidence of a substantial common genetic component to ALS risk, with SNP-based heritability estimates of 21 % [14]. The largest genome-wide association study (GWAS) of ALS (29,612 people with ALS and 122,656 controls) recently reported 15 genome-wide significant loci associated with ALS risk [15], replicating previous associations and identifying novel risk loci. The authors analysed each significant locus to highlight likely causal genetic variation using statistical fine-mapping and inferred the underlying molecular mechanisms through the integration of functional genomic annotations such as expression quantitative trait loci (eQTL) data.

Transcriptome-wide association study (TWAS), a powerful approach for the integration of eQTL data with GWAS summary statistics, infers up- and down-regulation of gene expression associated with the GWAS phenotype [16]. This approach provides valuable insights into the molecular mechanisms underlying genome-wide significant loci, and due to the aggregation of associations across variants and a reduced multiple testing burden, TWAS can identify significant associations within loci not previously achieving genome-wide significance in the GWAS [17,18]. A key advantage of TWAS over traditional studies analysing observed differential gene expression in cases and controls is the gain in power by combining the large sample sizes of GWAS with the precious and smaller eQTL datasets. This is particularly relevant for brain-related disorders for which the disease relevant tissue is only accessible post-mortem. The TWAS framework has recently been expanded to other molecular traits, such as altered protein-level data (pQTL), referred to as proteome-wide associations study (PWAS), uncovering unique insights into the downstream effects of disease associated loci [19].

TWAS and PWAS identify genes associated with the GWAS phenotype, but also provide the direction of effect on the phenotype, as well as some degree of spatiotemporal specificity depending on the samples used to derive TWAS/PWAS models [20]. This contrasts with functionally agnostic gene association analyses, such as MAGMA [21], which aggregate genetic associations within gene regions, thereby providing no information on direction of effect or spatiotemporal context. The additional information available from TWAS/PWAS can be used to enhance downstream analyses, such as drug repurposing analysis and prediction modelling.

Drug enrichment analysis using unsigned gene association summary statistics is a commonly used approach to identify drugs for repurposing [22]. However, no information regarding direction of effect is considered, so it is possible this approach might identify drugs that exacerbate pathological mechanisms underlying the GWAS phenotype. In contrast, TWAS/PWAS results can be used to provide insight into whether a given drug interacts with disease associated genes in a way that specifically reduce disease risk [23].

Another key application of GWAS is phenotype prediction, primarily using polygenic scores, calculated as the GWAS-effect size weights sum of associated alleles [24]. A limitation of polygenic scores is their limited predictive utility, typically only capturing a fraction of the SNP-based heritability. TWAS/PWAS results provide an alternative approach for phenotype prediction, as they can be used in combination with observed expression/protein data in a target sample to calculate omic-based scores predicting the GWAS phenotype [25,26]. When using TWAS results in combination with observed expression in a target sample, the resulting scores can be referred to as polytranscriptomic scores (PTS). PTS leverage the power of GWAS and capture both genetic and environmental risk an individual may carry, broadening the phenotypic variance that can be explained over polygenic scores.

We sought to further utilise the common genetic association results from the latest ALS GWAS, along with the latest functional genomic annotations. Here, we perform TWAS and PWAS of ALS to identify novel genetic associations with ALS, identify drug repurposing opportunities for ALS, and evaluate the predictive utility of PTS for prediction of ALS risk and clinical characteristics.

## 2 Methods

**Fig. 1:**
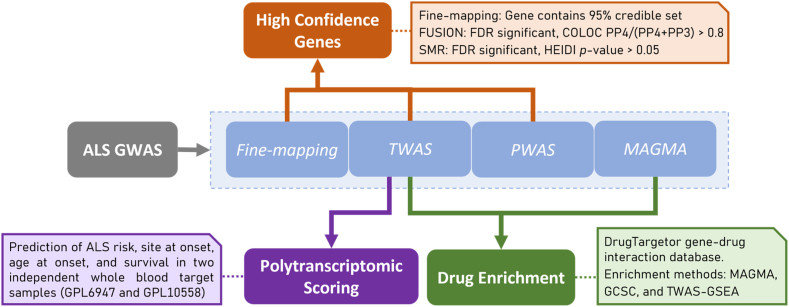
Schematic representation of study design and statistical analysis.

An overview of the study design is shown in Fig. 1. Using the latest ALS GWAS summary statistics, we performed a series of analyses to identify genes associated with ALS. Gene-finding analyses include SNP-based fine-mapping, integration of expression and protein data (TWAS and PWAS), and gene association analysis using MAGMA. The results of these analyses were used for three downstream aims. First, identification of high-confidence gene associations with ALS was achieved using results of SNP-based fine-mapping, TWAS and PWAS. Second, identification of novel drug repurposing opportunities for ALS was based on results from TWAS and MAGMA. Third, the results of TWAS were used to calculate PTS, which we then evaluated in two external blood expression ALS case-control datasets. More information regarding these analyses is provided below.

Analyses were performed using King's College London's High Performance Computing Cluster CREATE [27].

### 2.1 ALS GWAS summary statistics

We used the most recent ALS GWAS summary statistics, which are publicly available [15]. The European ancestry-only GWAS summary statistics were used to avoid linkage disequilibrium (LD) mismatch with external reference data downstream, all of which is derived within European populations. The European-only ALS GWAS included 27,205 people with ALS and 110,881 control participants. GWAS summary statistics underwent standard quality control (see Supplementary Information 1). GWAS summary statistics were restricted to variants with a minor allele frequency >1 %.

### 2.2 Gene finding analyses

#### 2.2.1 Fine-mapping

The SuSiE R package was used to perform variant-level statistical fine-mapping of ALS GWAS summary statistics [28]. SuSiE calculates the posterior inclusion probability of genetic variants being the casual variant for each genome-wide significant locus. SuSiE was performed assuming a single causal variant (L = 1) to avoid LD mismatch issues between the GWAS sample and 1 KG European reference population. As a sensitivity analysis, SuSiE was also performed allowing up to 10 independent causal signals. Further information is available in the Supplementary Information 2.

#### 2.2.2 TWAS/PWAS

##### 2.2.2.1 Expression and protein panels

Given ALS-associated genes are primarily expressed in brain tissues [15], we inferred differential expression/protein levels associated with ALS using a range of brain expression/protein panels (Table 1). Furthermore, we included blood expression panels, as these often have a larger sample size available than brain tissues, and blood eQTL data can be used as a proxy for brain tissues due to the moderate correlation between eQTL effects across tissues [29]. TWAS and PWAS were performed using FUSION software (https://github.com/gusevlab/fusion_twas). Most panels were previously derived and are publicly available via the FUSION website, though we generated novel TWAS models from post-mortem primary motor cortex RNA-seq and genotype data from the King's College London (KCL) and Medical Research Council London Neurodegenerative Diseases Brain Bank, herein referred to as ‘KCL Brain Bank’ [30,31]. KCL Brain Bank contains a mixture of people with ALS and controls, and FUSION software was used to generate the TWAS models after stringent quality control (see Supplementary Information 3).

**Table 1:** Expression and protein panels used for TWAS and PWAS.

Type	Software	Panel	N individuals	N genes
Expression	FUSION	Brain – Amygdala (GTEx)	119	2513
Expression	FUSION	Brain – Anterior cingulate cortex BA24 (GTEx)	135	3326
Expression	FUSION	Brain – Caudate basal ganglia (GTEx)	172	4884
Expression	FUSION	Brain – Cerebellar hemisphere (GTEx)	157	5885
Expression	FUSION	Brain – Cerebellum (GTEx)	188	7050
Expression	FUSION	Brain – Cortex (GTEx)	183	5425
Expression	FUSION	Brain – Frontal cortex BA9 (GTEx)	157	4388
Expression	FUSION	Brain – Hippocampus (GTEx)	150	3437
Expression	FUSION	Brain – Hypothalamus (GTEx)	156	3423
Expression	FUSION	Brain – Nucleus accumbens basal ganglia (GTEx)	181	4830
Expression	FUSION	Brain – Putamen basal ganglia (GTEx)	153	4175
Expression	FUSION	Brain – Substantia nigra (GTEx)	100	2191
Expression	FUSION	Brain – Spinal cord vervical c-1 (GTEx)	115	3033
Expression	FUSION	Blood (GTEx)	558	7832
Expression	FUSION	Brain – DLPFC (CMC)	452	5226
Expression	FUSION	Brain – DLPFC - Splice (CMC)	452	7771
Expression	FUSION	Blood (NTR)	1247	2437
Expression	FUSION	Blood (YFS)	1264	4699
Expression	FUSION	Brain – DLPFC (PsychENCODE)	1321	14750
Expression	FUSION	Brain – Motor cortex (KCL Brain Bank)	158	906
Expression	SMR	Brain – DLPFC (PsychENCODE)	1321	24560
Expression	SMR	Brain – Basalganglia (MetaBrain)	574	18406
Expression	SMR	Brain – Cerebellum (MetaBrain)	723	18417
Expression	SMR	Brain – Cortex (MetaBrain)	6601	18414
Expression	SMR	Brain – Hippocampus (MetaBrain)	206	18406
Expression	SMR	Brain – Spinalcord (MetaBrain)	285	18417
Expression	SMR	Blood (eQTLGen)	31684	19250
Protein	FUSION	Brain – DLPFC (ROSMAP)	376	1477
Protein	FUSION	Brain – DLPFC (Banner)	152	1148
Protein	SMR	Brain – DLPFC (ROSMAP)	376	7809

Note. SMR = Summary-based Mendelian Randomisation; GTEx = Genotype-Tissue Expression study; DLPFC = Dorsolateral Prefrontal Cortex.

##### 2.2.2.2 FUSION/SMR

We performed TWAS and PWAS using both FUSION and summary-based mendelian randomisation (SMR) methods to utilise the resources available for each of these methods and compare results across methods (see Supplementary Information 4). Both SMR and FUSION test whether genetic variants associated with the GWAS phenotype are also associated with differential gene expression or protein levels, using a subsequent colocalisation analysis to test whether the overlapping association is driven by the same causal variant. Further details can be found in Supplementary Information 4. False discovery rate (FDR) correction for multiple testing was performed across all expression panels for FUSION and SMR separately.

#### 2.2.3 MAGMA gene association analysis

MAGMA v1.10 was also used to calculate gene associations [21]. MAGMA estimates gene associations by calculating the mean association of variants within gene regions, accounting for LD using Brown's method. We defined gene locations using the NCBI37.3 locations file on the MAGMA website (https://ctg.cncr.nl/software/magma), using a 35 kb upstream and 10 kb downstream gene window, to allow for regulatory regions around each gene. We used the 1 KG Phase 3 European LD reference available on the MAGMA website.

### 2.3 Defining high-confidence genes

We used the following criteria to define genes associated with ALS with ‘high confidence’: Gene contains all variants within SuSiE 95 % credible set, FDR significant TWAS/PWAS association and colocalised (coloc software PP4/(PP3 + PP4) > 0.8), FDR significant SMR association and colocalised (HEIDI p-value >0.05). The threshold used to define colocalisation by coloc was chosen to highlight the probability that the association is driven by the same causal variant (PP4) rather than due to linkage (PP3). TWAS will have already determined an association in the locus for both traits and we can ignore the posterior probability of models where there is no association for either or both traits. Significant MAGMA gene associations are not considered high confidence as results are liable to confounding due to LD.

### 2.4 Drug repurposing analysis

#### 2.4.1 Enrichment methods

We tested for enrichment of drug-gene interactions using three approaches: MAGMA, Gene Co-regulation Score Regression (GCSC), and TWAS-based Gene Set Enrichment Analysis (TWAS-GSEA).

MAGMA gene-set enrichment analysis is based on MAGMA estimated gene associations and binary drug-gene interaction data [21]. GCSC regression is a method that leverages gene co-regulation to test for and enrichment of TWAS associations within gene-sets or associated with gene-properties [32]. TWAS-GSEA is based on TWAS estimated gene associations and directional drug-gene interaction data [33].

A key distinction between these methods is that MAGMA and GCSC do not account for the direction of association between the gene, the GWAS phenotype, and the drug. Therefore, when testing for an enrichment of genes that interact with a given drug, they fail to test whether the drug would decrease risk of ALS. In contrast, TWAS-GSEA does consider the direction of association between the gene, phenotype, and drug. As a result, enriched drugs using this method are suggested to induce expression changes reducing risk of ALS. Further details on these methods and their differences are provided in the Supplementary Information 5.

#### 2.4.2 Drug-gene interaction data

DrugTargetor is a tool for identifying drugs that interact with genes associated with a GWAS phenotype [22]. DrugTargetor uses a drug-gene interaction dataset drawn from a range of sources including ChEMBL, PHAROS, PDSP Ki database and NCBI PubChem BioAssay (https://github.com/hagax8/drugtargetor/blob/master/wholedatabase_for_targetor). The DrugTargetor dataset indicates whether a drug interacts with a given gene's expression or protein product, and whether the interaction leads to an increase or a decrease in activity. We used MAGMA, GCSC and TWAS-GSEA to test for enrichment of drugs based on the DrugTargetor drug-gene interaction dataset. Binary gene sets indicating drug-gene interactions were used for MAGMA and GCSC, as these methods do not account for the direction of effect. For TWAS-based enrichment, we coded drug-interactions as −1 or 1 to indicate whether the drug decreased (labelled as 'DECREASED_EXPRESSION', 'NEGATIVE_RESPONSE', or 'OPPOSITE_RESPONSE') or increased (labelled as 'INCREASED_EXPRESSION' or 'POSITIVE_RESPONSE') the activity of the gene. Otherwise, drug-gene interactions were coded as 0 where there was no directional evidence of drug-gene interaction. Drug enrichment analyses were restricted to drugs interacting with at least 2 genes with available association statistics.

#### 2.4.3 Anatomical therapeutic Chemical (ATC) enrichment analysis

After estimating enrichment of specific drugs, we tested whether drugs with specific level 3 ATC (pharmacological subgroup) codes were enriched. This was carried out using the non-parametric Wilcoxon test, testing whether drugs within a given ATC group were enriched for association compared to all other drugs available in DrugTargetor. A one-sided test was used for MAGMA and GCSC, but a two-sided test was used for TWAS-GSEA to test direction and significance of ATC group enrichments. FDR correction was used to account for multiple testing when determining statistical significance. ATC enrichment was restricted to ATC groups containing at least 5 drugs with drug enrichment statistics available.

### 2.5 Polytranscriptomic scoring (PTS)

#### 2.5.1 Target gene expression datasets

To assess the utility of PTS for predicting ALS risk and clinical features, we used two whole blood gene expression datasets from the Gene Expression Omnibus. These datasets are a part of the same Gene Expression Omnibus series (GSE112681) but separated due to the use of two array platforms to measure gene expression. The datasets corresponding to each platform are termed GPL6947 and GPL10558. These datasets both consist of people diagnosed with ALS and controls, and were originally used for a differential gene expression study of ALS [34]. The expression and phenotype data were downloaded using GEOquery. The expression data was averaged across probes for each gene using the limma R package ‘avereps’ function [35], and values were then log2 transformed. The following phenotypes were available for both datasets: ALS case-control status, site of onset (spinal vs bulbar), age at onset (years), and survival.

#### 2.5.2 Calculation of polytranscriptomic scores

An analogous approach to calculating polygenic scores can be used to calculate PTS. Polygenic scores are typically calculated as the sum of alleles weighted by their GWAS effect size. In contrast, PTS are calculated as the sum of observed expression values weighted by their TWAS effect size. Specifically, PTS were calculated as the sum of gene expression Z-scores, weighting each gene by the corresponding TWAS Z-score.

Analogous to the commonly used LD-based clumping and *p*-value thresholding for calculating polygenic scores, we accounted for the non-independence of nearby TWAS associations by clumping based on a predicted gene expression correlation matrix, and applied a range of *p*-value thresholds to select different numbers of genes to be included in the PTS. The predicted expression correlation matrix was generated by predicting expression into the European subset of the 1 KG reference sample using the SNP-weights in the corresponding TWAS models, and then calculating the Pearson correlation between all genes within a 500 kb window. We removed TWAS associations if they had a high correlation with a lead gene (*r*^*2*^ > 0.1). This clumping and thresholding approach was carried out using the IFRisk script (https://github.com/opain/Inferred-functional-risk-scoring) and is consistent with a previous study generating TWAS-based risk scores [36].

We generated PTS using TWAS results from all panels combined, and TWAS results from brain panels and blood panels separately. We also generated PTS using only TWAS associations that showed evidence of colocalisation (PP4 > 0.8). We only considered the colocalisation PP4 value to define colocalisation for this analysis as the PP4-PP3 ratio may be unstable for genes at lower TWAS p-value thresholds. We averaged TWAS Z-scores for a given gene if the gene was in multiple panels after clumping.

#### 2.5.3 PTS association analysis

We compared ALS PTS between people with ALS and healthy controls (binary) and compared the ALS PTS across clinical characteristics within people diagnosed with ALS alone, including spinal versus bulbar site of onset (binary), age at onset (continuous) and survival (continuous). We used linear regression to test the PTS association for all outcomes, converting the observed *R*^2^ to the liability scale for binary outcomes. For case-control analysis, we assumed a population prevalence of 1/300. For spinal-bulbar onset analysis, when converting to the liability scale we assumed an arbitrary population prevalence of 0.5 to aid comparison with future studies. We included sex as a covariate throughout.

We performed regression within each gene expression platform (GPL6947 and GPL10558) separately, and then meta-analysed results using inverse variance weighting.

#### 2.5.4 Observed differential expression analysis

In addition to TWAS, which infers differential expression associated with ALS risk, we also estimated the observed evidence of differential expression associated with ALS within the GPL6947 and GPL10558 ALS case-control cohorts. This was carried out to enable comparison of TWAS-inferred and observed differential expression, as this can highlight observed differential gene expression that is a consequence of the disease [26].

We tested the Pearson correlation between ALS case-control status and observed expression levels within the GPL6947 and GPL10558 cohorts separately, and subsequently meta-analysed using inverse-variance weighting. FDR correction was used to account for multiple testing when determining statistical significance.

## 3 Results

### 3.1 Gene discovery

We performed SNP fine-mapping, TWAS and PWAS based on ALS GWAS summary statistics to define a set of high-confidence genes associated with ALS.

**Fig. 2:**
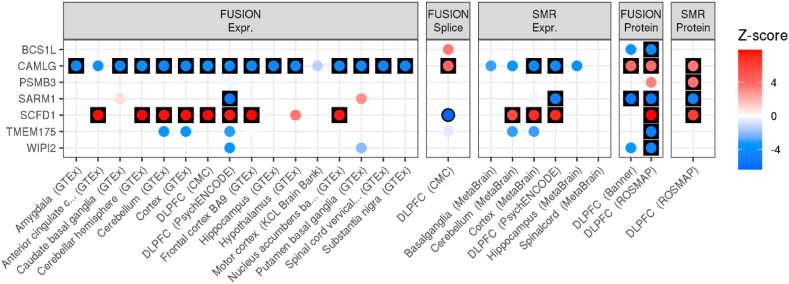
TWAS and PWAS associations for high-confidence ALS-associated genes defined using PWAS. Results are only shown for brain tissue expression and protein panels. Results are separated by the method and external data used. FUSION and SMR results are shown for all panels, with each point coloured according to the Z-score of association. Red indicates an increased expression/protein level in people diagnosed with ALS, and blue indicates decreased expression/protein level in people diagnosed with ALS. Results have a black outline if the association was FDR significant, and are in a black square if the association was FDR significant and showed evidence of colocalisation.

SNP-based fine-mapping, with an assumption of a single causal signal, identified five high-confidence genes (details in Supplementary Information 6 and tables S1–S2). TWAS using FUSION and SMR identified 108 genes with significant differential expression in ALS and strong evidence of colocalisation (tables S3–S4). PWAS using FUSION and SMR identified altered levels of 7 proteins in ALS and strong evidence of colocalisation, three of which were also identified as high confidence using TWAS (Fig. 2, T ables S5–S6). Across SNP fine-mapping, TWAS and PWAS analyses, 118 unique genes were identified as high-confidence associations. Further details of gene discovery results are in the Supplementary Information 6 and fig. S1.

### 3.2 Drug repurposing for ALS

**Fig. 3:**
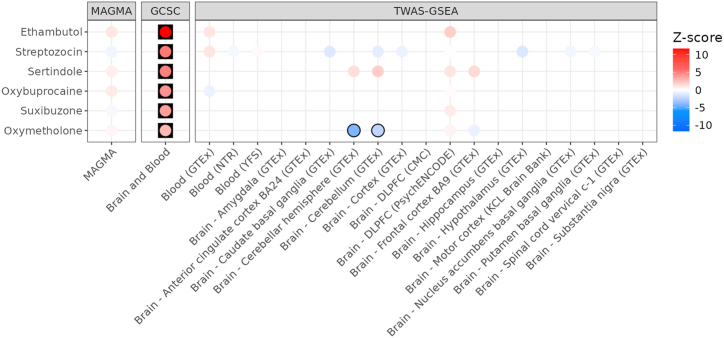
Drug enrichment results for ALS using MAGMA, GCSC and TWAS-GSEA. Each point is coloured according to the Z-score of enrichment. For TWAS-GSEA, positive Z-scores (red) indicates the drug reduces risk of ALS, whereas negative Z-scores (blue) indicate the drug increases risk of ALS. Results have a black outline if the association was nominally significant, and are in a black square if the association was FDR significant.

We used MAGMA, GCSC and TWAS-GSEA to test for enrichment of ALS-associated genes that interact with specific drugs (Fig. 3). GCSC identified six significantly enriched drugs, including Ethambutol (FDR = 3.84 × 10^−29^), Streptozocin (FDR = 5.39 × 10^−6^), Sertindole (FDR = 1.87 × 10^−5^), Oxybuprocaine (FDR = 3.04 × 10^−4^), Suxibuzone (FDR = 1.77 × 10^−3^), and Oxymetholone (FDR = 4.12 × 10^−2^). MAGMA and TWAS-GSEA did not identify any significantly enriched drugs after multiple testing correction. Of the drugs identified by GCSC, TWAS-GSEA found nominally significant evidence across two expression panels that Oxymetholone induces an expression signature that may increase risk of ALS. For the six drugs identified by GCSC, we list the interacting genes and their mechanism of action (T able S7).

**Fig. 4:**
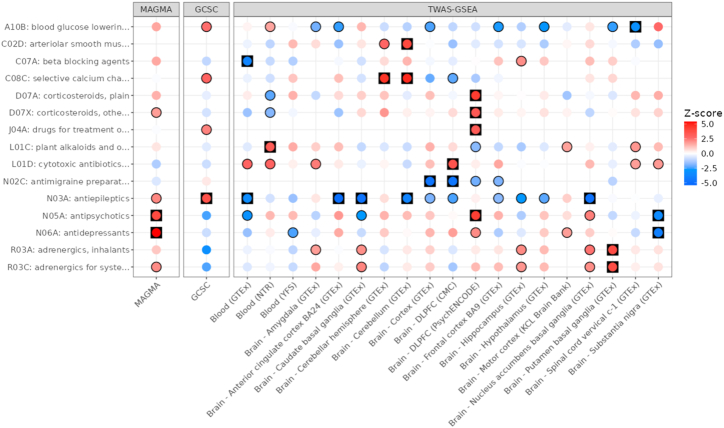
ATC enrichment for ALS based on results of MAGMA, GCSC and TWAS-GSEA. Each point is coloured according to the Z-score of enrichment. For TWAS-GSEA, positive Z-scores (red) indicate the drugs reduces risk of ALS, whereas negative Z-scores (blue) indicate the drugs increases risk of ALS. Results have a black outline if the association was nominally significant, and are in a black square if the association was FDR significant.

We then tested for enrichment of ATC classes based on the drug associations from MAGMA, GCSC and TWAS-GSEA (Fig. 4). Using MAGMA results identified 2 significant ATC categories, including ‘antidepressants’ (FDR = 3.55 × 10^−6^) and ‘antipsychotics’ (FDR = 3.02 × 10^−3^). Using GCSC results, only the ATC group ‘antiepileptics’ was significantly enriched (FDR = 0.015).

Using TWAS-GSEA results there were 22 FDR significant ATC enrichments across all panels, representing 15 unique ATC codes, 4 of which were significant across more than one expression panel. The most enriched ATC group reducing ALS risk was ‘selective calcium channel blockers with mainly vascular effects’, which was FDR significant using two expression panels. The most enriched ATC group increasing ALS risk was ‘antiepileptics’, FDR significant across five expression panels, consistent with enrichment results from GCSC. The ‘antipsychotics’ ATC group was significantly enriched using TWAS results from two panels, but the direction of enrichment was opposite: while antipsychotics associated with a decreased ALS risk based on PsychENCODE dorsolateral prefrontal cortex (DLPFC) data, they associated with an increased risk based on GTEx substantia nigra data. Other ATC groups enriched according to TWAS-GSEA are shown in Fig. 4.

#### 3.2.1 Prediction using polytranscriptomic scores (PTS)

**Fig. 5:**
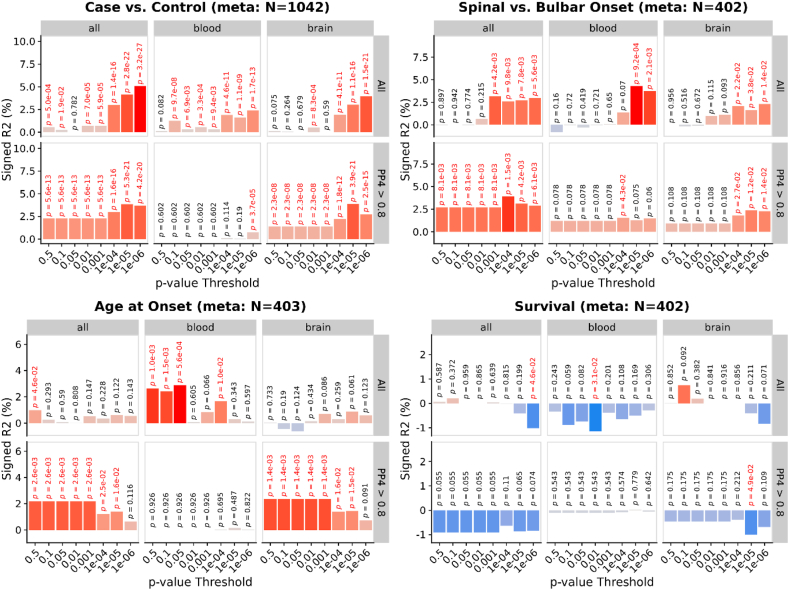
PTS association ALS risk and patient characteristics. Y-axis shows the percentage variance explained signed by the direction of association, on the liability scale for binary outcomes and on observed scale for continuous outcomes. P-values are shown above each bar, with nominally significant (p < 0.05) associations highlighted in red. Results are shown when deriving PTS using all TWAS panels, only blood panels and only brain panels. Results are also shown when deriving PTS using only TWAS associations that showed evidence of colocalisation (PP4 > 0.8).

Using inferred differential gene expression results from the ALS TWAS, we generated PTS in two ALS case-control target samples with observed whole blood expression, stratifying PTS by either brain or blood TWAS gene expression panels, and by evidence of colocalisation (PP4 > 0.8) (Fig. 5).

We found strong evidence that the ALS PTS can predict ALS case-control status (max. liability *R*^2^ = 5.1 %; *p*-value = 3.2 × 10^−27^). Within individuals with ALS, ALS PTS showed evidence of being associated with an increased likelihood of spinal onset ALS, a later age at onset, and decreased survival. There was no consistent effect of partitioning PTS to TWAS associations from either blood or brain expression panels, or by evidence of colocalisation. However, restricting to colocalised associations from blood-based TWAS results consistently reduced the predictive utility of the PTS. PTS association results for each target sample are separately shown in Supplementary Figs. 2–5. The number of genes in the PTS available in each target sample varied (T able S8).

### 3.3 Comparison of predicted and observed differential expression in ALS

We compared the predicted differential expression from TWAS to observed differential expression in the GPL6947 and GPL10558 ALS case-control blood expression cohorts (fig. S6). Of the 108 high-confidence genes identified using TWAS (FUSION or SMR), 44 genes were statistically significant in the observed differential expression analysis (fig. S6). Of the 36 high-confidence genes from TWAS using blood expression panels specifically, 18 genes had significant observed differential expression, with 8 genes showing a consistent direction of effect (*ATXN3, BTBD1, DHRS11, GGNBP2, MTAP, RNF24, TBK1*, *ZNHIT3)* and 10 genes showing a discordant direction of effect (*CAMLG*, *FNBP1*, *LY6G5C*, *NCR3*, *NDST2*, *PSMB10*, *PTBP2*, *SAR1B*, *SDHA*, *ZNF142*).

We also compared our list of high-confidence genes to results of previous studies examining single-cell transcriptomic and proteomic associations with ALS. Li et al. compared healthy controls to individuals with the *C9ORF72* repeat expansion and diagnosed with ALS [37]. Of the 85 high confidence genes present in the Li et al. results, 62 were reported as significant in at least one cell type (fig. S7). Comparison of direction of effect to our TWAS results is challenging as often the direction of association varied across cell types within a given tissue. Of the 36 high confidence genes that had a consistent direction of effect across cell types and tissues in the Li et al. study, 16 had the same direction of effect as reported in our TWAS analysis. Another study by Guise et al. compared protein levels in motor neurons between healthy controls and individuals diagnosed with ALS [38]. Two of the high confidence genes reported by our study, *AP3B2* and *SDHA*, were also reported as significant by Guise et al. The direction of effect of *AP3B2* in our TWAS was congruent with that observed by Guise et al., but the direction of effect of *SDHA* in our TWAS was discordant to that observed by Guise et al.

## 4 Discussion

This study has provided novel and mechanistic insights into the genes underlying ALS genetic risk. Furthermore, using these insights, we have identified drugs that could be used to treat ALS and derived an ALS PTS able to predict ALS risk. Collectively these results provide a key advance in our understanding of ALS aetiology, highlight novel research avenues for pharmacological treatment of ALS, and offer a new and non-invasive biomarker of ALS risk.

We used a combination of SNP fine-mapping, TWAS and PWAS analyses to define a set of high-confidence genes associated with ALS risk. By integrating eQTL and pQTL data we have uncovered novel molecular insights regarding ALS, aiding future experimental studies to further characterise the role of specific variants and genes in ALS. For example, we found that ALS risk leads to an increased expression of the *SCFD1* gene in brain tissues, which is consistent with a previous study [31], as well as inferring increased levels of the SCFD1 protein in brain tissues accompanied by a decreased expression of the *SCFD1* gene in the blood. 10.13039/100014337Furthermore, we infer ALS is associated with decreased expression of the *CAMLG* gene, supporting previous findings [39], which we expanded on by showing an increased level of the CAMLG protein in people with ALS. Further validation of this finding is required, but this discrepancy could be explained by post-transcriptional regulatory mechanisms, thereby decoupling the relationship between expression and protein levels. For example, the ALS risk variant could increase the post-translational stability of the CAMLG protein, leading to reduced expression of the gene due to a negative feedback loop. Our study also identified many novel ALS-associated genes, such as *TMEM175*. Our study highlights genetic risk for ALS confers a reduced level of the TMEM175 protein in the DLPFC. TMEM175 is a lysosomal ion channel, previously linked to Parkinson's disease, and a deficiency of TMEM175 has been found to cause decreased mitochondrial respiration, altered lysosomal pH and impaired autophagy [40]. Together these findings suggest TMEM175 deficiency may also increase risk of ALS, providing a new insight into the aetiology of ALS.

We then explored several strategies for the identification of drugs that could be repositioned for treating ALS. We leveraged the molecular insights from our TWAS analysis to identify drugs and drug classes that interact with differentially expressed genes in people with ALS, and furthermore indicate whether these drugs might reverse the ALS-risk associated expression profile. Using the method GCSC, we identified six drugs that are enriched for interactions with genes differentially expressed in people with ALS. However, GCSC does consider directional information and cannot infer whether these drugs would have a therapeutic or deleterious effect in ALS. There was insufficient power for directional drug repurposing analysis using TWAS-GSEA to provide robust insights, so further investigation of the effect of these drugs in ALS is required. One of the enriched drugs, suxibuzone, is a nonsteroidal anti-inflammatory drug (NSAID), which as a group have been implicated for the treatment of ALS [41], warranting further investigation of suxibuzone specifically. Another enriched drug was sertindole, an atypical antipsychotic with a strong affinity for dopamine D2, serotonin 5-HT2A, serotonin 5-HT2C, and alpha-1-adrenergic receptors [42]. It has been found to trigger autophagy in neuronal cells, which can lead to cell death. Further research is needed to understand the potential therapeutic process induced by sertindole. Oxymetholone, an anabolic steroid, was also enriched according to GCSC, but directional analysis using TWAS-GSEA showed nominally significant evidence that the drug might exacerbate the ALS pathology. Although ALS risk has not been linked to Oxymetholone, this finding is supported by previous research showing another anabolic steroid (Nandrolone) exacerbates disease pathology in *SOD1* mouse models of ALS [43,44]. Given these findings, further investigation of the effects of Oxymetholone, and other anabolic steroids, on ALS pathology is warranted. While conducting our study, another study was published utilising the latest ALS GWAS summary statistics and TWAS methodology to infer drug repurposing opportunities for ALS [45]. This previous study highlights five drugs as repurposing candidates, none of which were implicated in our study. However, the previous study used a distinct approach, manually identifying drugs interacting with individual ALS genes, in contrast with our statistical approach which considers the drug interactions with multiple genes when identifying drug repurposing candidates.

Beyond enrichment of individual drugs, our analyses provided a broader insight into which classes of drugs might be relevant to ALS. The most strongly enriched drug class predicted to have therapeutic benefits for ALS was selective calcium channel blockers, drugs primarily used to treat hypertension. Several studies show an association between hypertension and ALS, with a recent mendelian randomisation study supporting a causal role between prescription of calcium channel blockers and reduced risk of ALS [46], supporting the results of this study and highlighting a novel therapeutic avenue for ALS. In addition, we identified antiepileptic drugs were strongly enriched for interactions with ALS associated genes, however their direction of effect is predicted to exacerbate ALS risk. This finding is novel and should be further investigated to understand the implications for our understanding of ALS aetiology. One antiepileptic drug (ezogabine) has been found to have potential therapeutic effects on ALS [47], so further investigation of which antiepileptics might increase risk for ALS is needed. Interestingly, the antipsychotics drug class was significantly enriched in two tissues, but with opposite directions of effect. This could suggest that antipsychotic drugs interact with tissue-specific differential expression associated with ALS. However, comparisons across tissues should be made cautiously, as differences in reference data used for TWAS across tissues may contribute to inconsistencies. In this study, we prioritize findings that are consistent across tissues.

To further utilise our novel ALS differential expression results from TWAS, we combined our results with observed blood expression data from people diagnosed with ALS and controls to calculate PTS. We then evaluated whether PTS could predict ALS risk and clinical characteristics. Looking across two independent target expression datasets, the association between ALS PTS and case-control status was highly significant, explaining up to 5.1 % of ALS risk on the liability scale. This suggests blood-based PTS can provide a key advance in our ability to diagnose and predict ALS risk and could be used in combination with other risk factors as a non-invasive clinical biomarker of ALS risk. Furthermore, our study showed evidence that the PTS was associated with a spinal site of onset, later age at onset and decreased survival, suggesting ALS PTS might help guide the prognosis of people with ALS. These findings indicate differential expression inferred by TWAS is concordant with differential expression observed in people with ALS and controls associated with ALS. However, the target sample expression data was collected after diagnosis of ALS and possibly pharmacological treatment, which may confound our PTS association results. Therefore, further research regarding the predictive utility of ALS PTS in other contexts is required. Nonetheless, these findings support the predictive utility of PTS, congruent with previous studies showing PTS significantly predict Crohn's disease [26] and attention deficit hyperactivity disorder (ADHD) [25].

In addition to evaluating the predictive utility of the PTS, we contrasted the genetically-inferred differential expression from TWAS with *observed* differential expression in the ALS case-control gene expression datasets. Many genes were identified as significantly associated with ALS risk using both approaches, though a large proportion showed discordant directions of effect. A similar mixture of concordant and discordant effects was also observed when comparing to previously published single-cell based studies of ALS. Observed differential expression associated with ALS may reflect the gene's causal role in ALS pathology, but differential expression within people with ALS may also be a consequence of the disease. Given genetically-inferred differential expression is not susceptible to reverse causation, discordance between inferred and observed expression may help distinguish the molecular mechanisms by which these associations occur. A previous study regarding Crohn's disease demonstrated genes showing discordant genetically-inferred and observed differential expression are more likely to be triggered in response to immune stimuli [26]. A possible example of this from our study is the gene *SAR1B, which* was inferred to be downregulated in those with ALS using TWAS but was observed to upregulated in those with ALS. This could indicate that *SAR1B* is upregulated in those with ALS as a protective response to ALS pathology, and genetic variation in this locus is associated with ALS because it reduces the extent to which *SAR1B* can be upregulated. *SAR1B* has been reported to provide protection against inflammatory processes [48], consistent with the notion that *SAR1B* upregulation is a protective response to ALS pathology.

While conducting this study, two other studies were published using PWAS to infer proteomic associations with ALS risk [49,50]. The PWAS analyses by Gu et al. and Ma et al. were based on highly overlapping data sets used in our study, and as expected, the results are highly concordant. However, our study provides several key advances over these previous studies. Firstly, neither the Gu et al. or Ma et al. studies perform drug repurposing analysis or polytranscriptomic score analyses. These are two important advances in the field of ALS research, offering much needed insights into the treatment and prediction of ALS. Secondly, our study provides a more comprehensive comparison of TWAS and PWAS results across tissues, providing higher resolution information for follow up studies. We included novel gene expression reference data from the motor cortex, a tissue of particular interest when studying ALS. While Gu et al. did compare PWAS and TWAS results from the DLPFC, they did not report that the *CAMLG* association had a different direction of effect in the TWAS and the PWAS, despite this being shown in supplementary tables. Ma et al. compared results from their PWAS to observed differential expression results from several tissues but did not perform TWAS across tissues, making it difficult to interpret discordant PWAS and observed expression results.

Our study should be considered in the context of several limitations. First, in this study we only consider *common* genetic variation associated with ALS. However, the genetic architecture of ALS includes a combination of common and rare genetic variation. Considering the implicated genes and pathways from both sources of ALS risk will provide further insight into possible therapeutic avenues and improve risk prediction. Second, we only consider ALS GWAS summary statistics and functional genomic annotation data based on European ancestry individuals. The expansion of ALS and functional genomic studies in diverse populations will improve the generalisability of the prediction models across populations, as well as strengthen insights into causal mechanisms. Third, although large advances have been made in developing functional genomic datasets, such as eQTL studies, the current samples sizes prohibit the use integration of distal regulatory elements (e.g., trans eQTLs). Furthermore, the eQTL and pQTL datasets used in this study are based on bulk tissue samples, and integration of single cell-based panels in the future will enable further mechanistic understanding of ALS [51].

In conclusion, this study provides novel mechanistic insights into the genes associated with ALS, drug enrichment analysis has highlighted several therapeutic research avenues, and our findings indicate PTS may be a powerful predictor of ALS risk.

## Funding

OP is supported by a Sir Henry 10.13039/100004440Wellcome Postdoctoral Fellowship [222811/Z/21/Z]. The funders had no role in study design, data collection and analysis, decision to publish, or preparation of the manuscript. AI is funded by 10.13039/100009362South London and Maudsley NHS Foundation Trust, 10.13039/100011776MND Scotland, 10.13039/501100000406Motor Neurone Disease Association, 10.13039/501100000272National Institute for Health Research, 10.13039/100017026Spastic Paraplegia Foundation, 10.13039/501100000833Rosetrees Trust and Darby Rimmer MND Foundation. Funding for open access charge: 10.13039/100014013UKRI. AAC is an NIHR Senior Investigator (NIHR202421). This is in part an EU Joint Programme - Neurodegenerative Disease Research (JPND) project. The project is supported through the following funding organisations under the aegis of 10.13039/100013278JPND - www.jpnd.eu (United Kingdom, 10.13039/501100000265Medical Research Council (MR/L501529/1; MR/R024804/1)). This study represents independent research part funded by the 10.13039/501100000272National Institute for Health Research (10.13039/501100000272NIHR) Biomedical Research Centre at 10.13039/100009362South London and Maudsley NHS Foundation Trust and King's CollegeLondon. OP, AI and 10.13039/501100023375AAC obtained additional financial support for this study from the Turing Institute. JK was supported by the Polish National Science Centre [2020/37/B/NZ2/03268]. AAK is funded by 10.13039/100000971ALS Association Milton Safenowitz Research Fellowship (grant number22-PDF-609.10.13039/100000201DOI:10.52546/pc.gr.150909." title = "doi:10.13039/100000201DOI:10.52546/pc.gr.150909.">10.13039/100000201DOI:10.52546/pc.gr.150909.), The 10.13039/501100000406Motor Neurone Disease Association (10.13039/501100000406MNDA) Fellowship (Al Khleifat/Oct 21/975-799), The Darby Rimmer Foundation, and The 10.13039/100019418NIHR Maudsley Biomedical Research Centre. JMR is supported by Alzheimer's Research UK and the 10.13039/100012338Alan Turing Institute/10.13039/501100000266Engineering and Physical Sciences Research Council (EP/N510129/1). DJL is supported by Alzheimer's Research UK, National Institute for Health Research (NIHR) Applied Research Collaboration (ARC) South West Peninsula, 10.13039/501100000925National Health and Medical Research Council (10.13039/501100000925NHMRC), 10.13039/100000049National Institute on Aging/10.13039/100000002National Institutes of Health (RF1AG055654), and the 10.13039/100012338Alan Turing Institute/10.13039/501100000266Engineering and Physical Sciences Research Council (EP/N510129/1). 10.13039/100016174AJ was funded by the 10.13039/501100000406MND Association grant number Jones/Oct 15/958-799.

This work was facilitated by the NEUROHACK 2022 hackathon, organized by the Deep Dementia Phenotyping (DEMON) 10.13039/100031212Network, with additional financial support from the 10.13039/100012338Alan Turing Institute, Alzheimer's Research UK, the 10.13039/501100000406Motor Neurone Disease Association (10.13039/501100000406MNDA), and 10.13039/100012357LifeArc.

This research was funded in whole or in part by the 10.13039/100010269Wellcome Trust [222811/Z/21/Z]. For the purpose of open access, the author has applied a CC-BY public copyright licence to any author accepted manuscript version arising from this submission.

## Data availability statement

Data included in article/supp. material/referenced in article.

## CRediT authorship contribution statement

**Oliver Pain:** Writing – review & editing, Writing – original draft, Funding acquisition, Formal analysis, Data curation, Conceptualization. **Ashley Jones:** Writing – review & editing, Resources, Data curation, Conceptualization. **Ahmad Al Khleifat:** Writing – review & editing, Supervision, Data curation, Conceptualization. **Devika Agarwal:** Writing – review & editing, Methodology, Conceptualization. **Dzmitry Hramyka:** Writing – review & editing, Methodology, Conceptualization. **Hajer Karoui:** Writing – review & editing, Methodology, Conceptualization. **Jędrzej Kubica:** Writing – review & editing, Methodology, Conceptualization. **David J. Llewellyn:** Writing – review & editing, Conceptualization. **Janice M. Ranson:** Writing – review & editing, Conceptualization. **Zhi Yao:** Writing – review & editing, Conceptualization. **Alfredo Iacoangeli:** Writing – review & editing, Supervision, Resources, Methodology, Data curation, Conceptualization. **Ammar Al-Chalabi:** Writing – review & editing, Supervision, Funding acquisition, Data curation, Conceptualization.

## Declaration of competing interest

The authors declare the following financial interests/personal relationships which may be considered as potential competing interests: OP provides consultancy services for UCB pharma company. AAC reports consultancies or advisory boards for Amylyx, Apellis, Biogen, Brainstorm, Cytokinetics, GenieUs, GSK, Lilly, Mitsubishi Tanabe Pharma, Novartis, OrionPharma, Quralis, and Wave Pharmaceuticals. The other authors declare no competing interests.
